# Not all the bowlegs is rickets! (a case report)

**DOI:** 10.11604/pamj.2022.42.161.33990

**Published:** 2022-06-29

**Authors:** Keta Vagha, Patel Zeeshan Jameel, Jayant Vagha, Ashish Varma, Siddhartha Murhekar, Parameshwar Reddy, Spandana Madirala

**Affiliations:** 1 Department of Pediatrics, Jawaharlal Nehru Medical College, Sawangi (Meghe), Wardha, Maharashtra, India,; 2 Department of Urosurgery, Jawaharlal Nehru Medical College, Sawangi (Meghe), Wardha, Maharashtra, India

**Keywords:** Bowing, Blount’s disease, tibial intorsion, case report

## Abstract

Bowing of the legs is common in childhood. Most times it is considered to be rickets without considering other possibilities. Blount´s disease is a close differential diagnosis which is developmental deformity characterized by intorsion of tibia leading to varus angulation. This case report aims to encourage pediatricians to expand their vision and consider other possibilities when a case of bowing of legs is encountered. Here we report a case of a four-year-old boy with bowing of both legs noticed first at 2.5 years of age. There was no history suggestive of trauma. Development of the child was age appropriate in all domains. He was receiving treatment for rickets for 1.5 years in form of oral vitamin D3 and calcium supplementations. He had no other clinical signs of rickets like frontal bossing, widening of wrists, and rachitic rosary except bowing of legs. His biochemical parameters did not show any alterations that would support the diagnosis of rickets. Weight-bearing radiographs of lower limbs showed medial intorsion of bilateral tibia with metaphyseo-diaphysial angle to be 25º on the right side and 20º on the left side, which was beyond the physiological normal angulation, therefore he was diagnosed as a case of Blount´s disease, stage III as per Langenskiöld classification. All the bow legs is not always rickets in pediatric practice. Therefore, various differential diagnoses should be kept in mind as early diagnosis and intervention can change a child´s life.

## Introduction

Bowing of the legs is common during childhood. In majority of the children with bowlegs, it is the physiological variation in the normal growth pattern [1], but there are certain cases where these angular deformities are beyond the physiological variation. In rural India, there is a tunnel mindset regarding bowing of legs and is always considered to be Rickets. This case report aims to encourage the pediatricians to broaden the mindset and consider other possibilities when a case of bowing of legs is encountered. In this case report, we report a case of a four-year-old boy with infantile Blount´s disease.

## Patient and observation

**Information of the patient**: a four-year-old boy presented with bowing of both legs noticed first when he was 2.5 years old by parents. There was no history suggestive of trauma. He was treated for rickets for 1.5 years in form of oral vitamin D3 and calcium supplementations. Birth history was uneventful. There was no relevant family history. Development of the child was age appropriate in all domains. He started walking without support at around 15 months of age.

**Figure 1:**
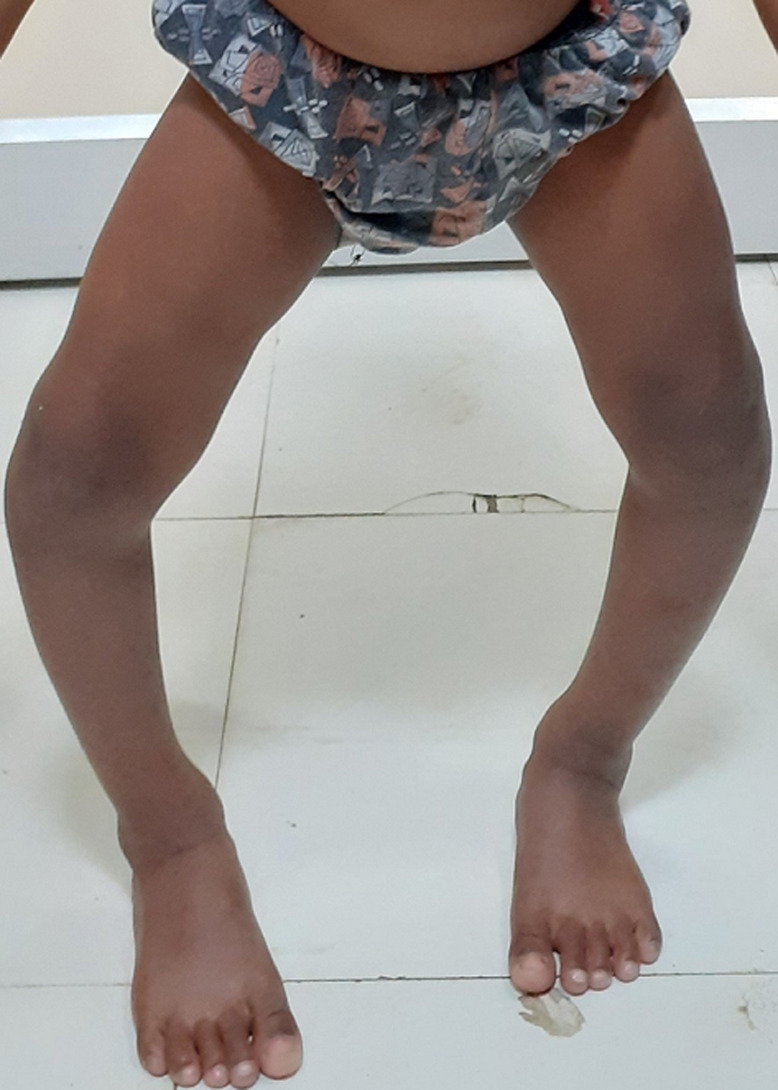
clinical photograph showing extensive bowing of bilateral lower limbs

**Clinical results**: on examination, he was short statured with height of 92 cm (<-3 SD), weight of 13 kg (-2 to -3 SD) and upper to lower segment ratio of 1.4: 1. He had normally shaped head with no facial dysmorphism. In addition, no clinical signs of rickets like frontal bossing, widening of wrists, and rachitic rosary except bowing of legs were present (Figure 1). Rest of the systemic examination was within normal limits.

**Figure 2:**
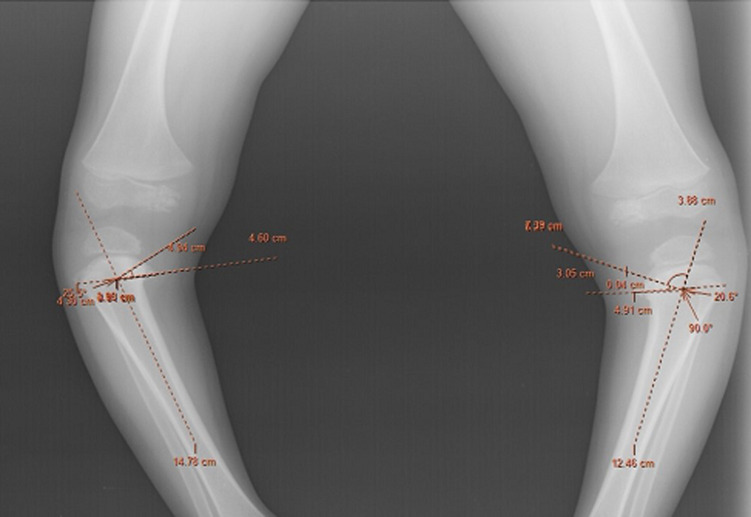
weight-bearing radiographs of lower limbs showing medial intorsion of bilateral tibia with metaphyseo-diaphysial angle to be 25º on the right side and 20º on the left side

**Figure 3:**
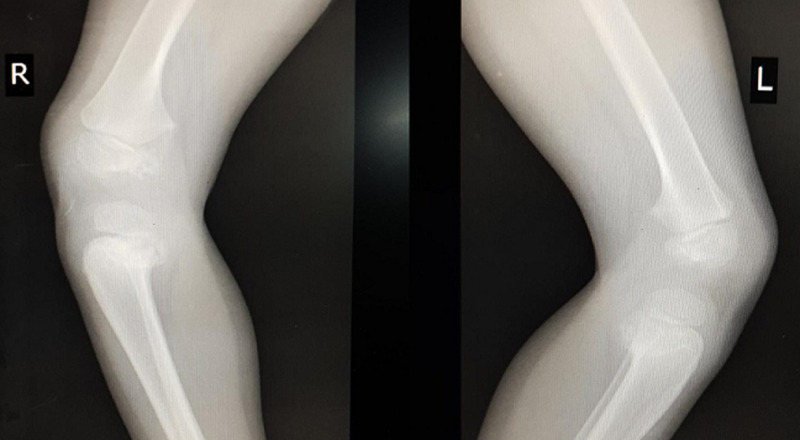
lateral view of radiographs of knee joint showing posterior projection of proximal tibial metaphysis

**Diagnostic approach**: his investigative blood panel showed a normal hemogram profile. Renal functions were normal. Further, the biochemical parameters showed a total calcium of 10.4 mg/dl with ionic calcium 4.8 mg/dl, serum phosphorus of 6.1 mg/dl and a normal serum alkaline phosphatase (ALP) of 236 IU/L. Radiographs of lower limbs showed medial intorsion of bilateral tibia with metaphyseo-diaphysial angle of 25º on the right side and 20º on the left side which was beyond the physiological normal angulation (Figure 2). Therefore, he was diagnosed as a case of Blount´s disease, stage III as per Langenskiöld classification. The lateral view of radiographs of knee joint showed posterior projection of proximal tibial metaphysis (Figure 3).

**Therapeutic interventions**: the child was then referred to Department of Pediatric Orthopedics for surgical management. He underwent oblique tibial osteotomy with an uneventful post-operative period.

**Figure 4:**
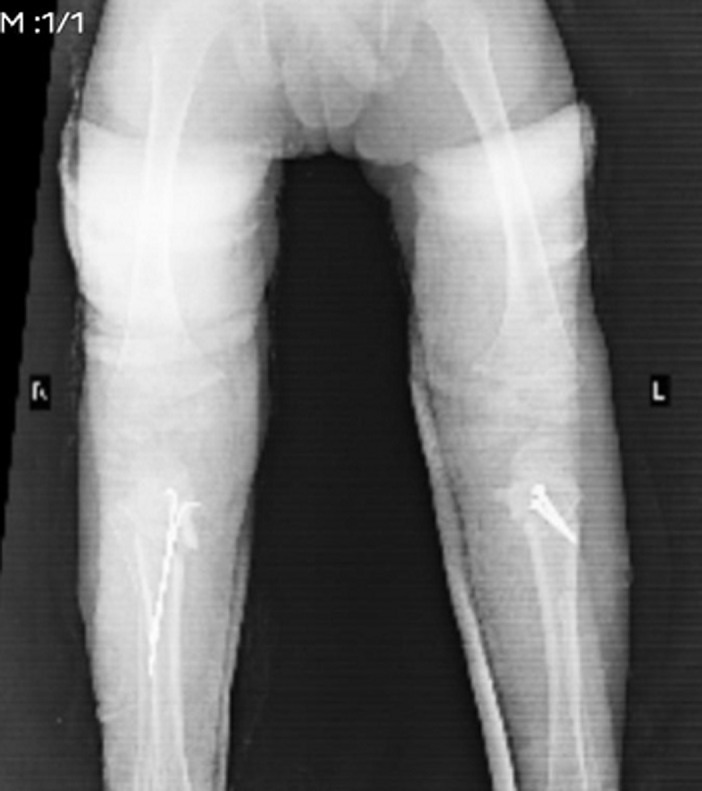
post-operation radiograph showing significant correction in bowing of legs

**Follow-up and outcome of interventions**: on follow-up, a significant correction in bowing was observed (Figure 4) with much improved gait and mobility. The child continued to receive physiotherapy and there was significant improvement in the walking due to which the child could perform his daily activities without any difficulty.

**Informed consent**: written informed consent was obtained from the patient´s parents for publication of this case report and any accompanying images and videos. A copy of the written consent is available for review by the Editor-in-Chief of this journal.

## Discussion

Bowing of legs in children, especially less than two years of age, is predominantly physiological and corrects as the child grows older. In comparison, pathological bowing tends to worsen with time if not corrected early and may ultimately result in a permanent morbidity. Thus, a pediatrician´s role is vital in early identification and appropriate management of bowing of legs. An important cause of bowing of leg is Blount´s disease, also known as “Osteochondrosis Deformans Tibiae” or “Tibia Vara” [2], a close differential to bowing of legs seen secondary to rickets [2,3]. Blount´s disease is a developmental deformity characterized by intorsion of tibia leading to varus angulation which is due to growth plate disorder of the medial aspect of the proximal tibial physis [4]. The etiopathogenesis of this disease is still unknown.

A multifactorial origin comprising of racial and genetic predisposition, obesity or early walking age contributing to increased mechanical pressure on the growth plate and probably the nutritional status of the child as well [5]. There are three distinct forms of Blount´s disease depending upon the presenting age group. The infantile form is seen in children aged between 1-4 years, juvenile form manifests late, after 4 years of age while the adolescent form is seen after 10 years of age. Clinically, the child has a variable degree of varus deformity of the proximal tibia, tibial intorsion and “beaking” of the proximal medial tibial epiphysis and metaphysis. In unilateral cases, limb length discrepancy may also be seen. On examination, there is no tenderness, or joint effusion or any restriction in degree of joint movement. However, some degree of gait instability may be present. As the child walks a characteristic lateral thrust may be seen [3]. Radiologically, Langenskiöld classified this disease into 6 progressive stages depending upon the epiphyseal-diaphyseal-metaphyseal distortion. Differential diagnosis includes physiological bowing, rickets secondary to dietary vitamin D deficiency or vitamin D resistant rickets, skeletal dysplasia and osteogenesis imperfecta (Table 1) [6-8]. Physiological bowing is generally subtle and symmetric, with the metaphyseo-diaphyseal angle being less than 11 degrees. While in Blount´s disease, the bowing is asymmetric, abrupt, and as sharp angulation with metaphyseo-diaphyseal angle more than 11 degrees.

**Table 1:** features of differential diagnosis

	Physiologic bowing [6]	Rickets [6]	Blount´s disease [6]	Skeletal dysplasia [7]	Osteogenesis imperfecta [8]
**Clinical features**	**-**Common torsional condition secondary to normal in**-**utero positioning. **-**Spontaneous resolution with normal growth and development.	**-**General **-**Failure to thrive (malnutrition) **-**Listlessness **-**Protruding abdomen **-**Muscle weakness (especially proximal) **-**Fractures (pathologic, minimal trauma) **-**Head: Craniotabes, frontal bossing, delayed fontanel closure, craniosynostosis **-**Chest: Rachitic rosary, Harrison´s groove **-**Back: Scoliosis, kyphosis, lordosis **-**Extremities: Enlargement of wrists and ankles, Valgus or varus deformities, Windswept deformity (valgus deformity of one leg with varus deformity of other leg), anterior bowing of tibia and femur, coxa vara, leg pain	**-**Abnormal endochondral ossification of the medial aspect of the proximal tibial physis leading to varus angulation of the tibia. **-**Infantile form: most common, bilateral with prominent medial metaphyseal beak, internal tibial torsion and leg length discrepancy. **-**Juvenile and adolescent forms: normal or greater than normal height, no palpable proximal medial metaphyseal beak, minimal internal tibial torsion, mild medial collateral ligament, ligament laxity, mild lower extremity length discrepancy.	**-**Disproportionately short stature **-**Delayed motor milestones. **-**Pain, deformity and neural deficits like paraparesis and quadriparesis. **-**Large head with hydrocephalus **-**Bowlegs with waddling gaits. **-**Atlantoaxial instability. **-**Cervical or thoracolumbar kyphosis.	**-**multiple repeated or unexplained fractures **-**short stature **-**kyphosis **-**scoliosis **-**discrepancy in length of lower limbs and bowing **-**blue sclerae **-**greyish or yellowish teeth **-**skin fragility **-**joint and ligament hyperlaxity **-**Early hypoacusia
**Radiological findings**	**-**Gentle and symmetric deformity. **-**Metaphyseal**-**diaphyseal angle <11 degrees. **-**Normal appearance of proximal tibial growth plate **-**No significant lateral thrust.	**-**Rachitic changes at wrist **-**Meatphysis shows fraying and cupping. **-**Widening of distal end of metaphysis. **-**Coarse trabeculation of the diaphysis. **-**Generalised rarefaction	**-**Asymmetric, abrupt and sharp angulation. **-**Metaphyseal**-**diaphyseal angle >11 degrees. **-**Medial sloping of the epiphysis. **-**Widening of the physis. **-**Fragmentation of the metaphysis **-**Significant lateral thrust.	**-**Disproportional shortening **-**oval translucent area in proximal femora and humeri **-**Dumbbell shaped appearance of long bones **-**Bowing of limbs **-**Calcified projections at lateral femoral metaphyses **-**cupping at the ends of ribs and long bones and metaphyseal flaring **-**long bone fractures **–**cone-shaped epiphyses **-**Proximal pointing of metacarpals **-**stippling of epiphyses **-**polydactlyly **-**duplicated calcaneus	**-**cortical bone thinning and excessive trabecular bone transparency **-**multiple fractures **-**multiple wormian bones in skull **-**diaphyseal bending or angulation **-**hyperplastic callus formation **-**ossification of interosseous membranes **-**popcorn calcifications **-**dense metaphyseal bands

Early diagnosis and treatment helps in correcting the deformity and having near normal limbs. The treatment depends upon the stage of the disease as classified by Langenskiöld. The children belonging to Langenskiöld stage <3 and below 3 years, are managed conservatively with braces with trial period of one year. Children with Langenskiöld stage >3 and more than 3 years or those with severe deformity undergo surgical intervention in form of valgus osteotomy and associated fibular diaphyseal osteotomy. In our case as well, at the first glance of the child, this seemed like a clear-cut case of vitamin D deficiency rickets. However, the absence of radiological findings of rickets as well as the normal calcium, vitamin D level, phosphorus and serum ALP levels prompted us to consider alternative diagnoses. The child underwent a successful oblique tibial osteotomy with good post-operative outcomes in terms of decreased degree of varus as well as improved mobility. Therefore, in cases with less obvious clinical signs and biochemical parameters, Blount´s disease should always be kept in mind. Certain clues for Blount´s disease are overweight child, walking early in life or affected family member.

## Conclusion

All the bowlegs is not always rickets in pediatric practice. Therefore, various differential diagnosis should be kept in mind as early diagnosis and intervention can change a child´s life by avoiding the progressive worsening of the deformity and severe articular distortion which may result in premature osteoarthritis of knees [9].
